# Malignant Pleural Mesothelioma: State-of-the-Art on Current Therapies and Promises for the Future

**DOI:** 10.3389/fonc.2019.01519

**Published:** 2020-01-24

**Authors:** Fabio Nicolini, Martine Bocchini, Giuseppe Bronte, Angelo Delmonte, Massimo Guidoboni, Lucio Crinò, Massimiliano Mazza

**Affiliations:** ^1^Biosciences Laboratory, Istituto Scientifico Romagnolo per lo Studio e la Cura dei Tumori (IRST) IRCCS, Meldola, Italy; ^2^Department of Medical Oncology, Istituto Scientifico Romagnolo per lo Studio e la Cura dei Tumori (IRST) IRCCS, Meldola, Italy; ^3^Immunotherapy and Cell Therapy Unit, Istituto Scientifico Romagnolo per lo Studio e la Cura dei Tumori (IRST) IRCCS, Meldola, Italy

**Keywords:** malignant pleural mesothelioma (MPM), immunotherapy, mesothelin, CAR (chimeric antigen receptor) T cells, miRNA replacement

## Abstract

Malignant pleural mesothelioma (MPM) is a rare, aggressive cancer of the pleural surface associated with asbestos exposure. The median survival of MPM patients is a mere 8–14 months, and there are few biomarkers and no cure available. It is hoped that, eventually, the incidence of MPM will drop and remain low and constant, given that most nations have banned the use of asbestos, but in the meantime, the incidence in Europe is still growing. The exact molecular mechanisms that explain the carcinogenicity of asbestos are not known. Standard therapeutic strategies for MPM include surgery, often coupled with chemotherapy and/or radiotherapy, in a small percentage of eligible patients and chemotherapy in tumors considered unresectable with or without adjuvant radiotherapy. In recent years, several new therapeutic avenues are being explored. These include angiogenesis inhibitors, synthetic lethal treatment, miRNA replacement, oncoviral therapies, and the fast-growing field of immunotherapy alone or in combination with chemotherapy. Of particular promise are the multiple options offered by immunotherapy: immune checkpoint inhibitors, tumor vaccines, and therapies taking advantage of tumor-specific antigens, such as specific therapeutic antibodies or advanced cell-based therapies exemplified by the CAR-T cells. This review comprehensively presents both old and new therapeutic options in MPM, focusing on the results of the numerous recent and on-going clinical trials in the field, including the latest data presented at international meetings (AACR, ASCO, and ESMO) this year, and concludes that more work has to be done in the framework of tailored therapies to identify reliable targets and novel biomarkers to impact MPM management.

## Introduction

Malignant pleural mesothelioma (MPM) is a rare, incurable, aggressive cancer of the pleural surface associated with asbestos exposure with a median survival of 8–14 months (1, 2). Although the incidence in some countries, e.g., the USA (3,200 cases/year) (3), is fairly constant, in Europe, it is growing and is expected to peak between 2020 and 2025 (1). Moreover, the migratory phenomena toward western countries from nations lacking legislation on asbestos use will render MPM even more frequent. At present, no actionable driver mutations have been identified in MPM. However, MPM carcinogenesis and outcome are influenced by many factors: BRCA-associated protein 1 (BAP1) expression status, CDKN2A and neurofibromatosis type 2 (NF2) tumor suppressor inactivation, overexpression of growth factors such as vascular endothelial growth factor (VEGF), mesothelin (MSLN) promoter methylation, and Ras/mitogen-activated protein kinase and phosphatidylinositol 3-kinase/mTOR pathway activation (4, 5).

## Mesothelioma Therapies

### Standard

The standard therapeutic strategies for MPM are (i) surgery for resectable tumors, often combined with radiotherapy (RT) and/or chemotherapy (CT) (trimodality treatment), and (ii) CT or RT in unresectable tumor cases. To date, the only FDA- and EMA-approved frontline therapy is the cisplatin-pemetrexed combination (6–10). Only selected patients can benefit from a complete resection, either lung-sacrificing surgery (extrapleural pneumonectomy, EPP) or lung-sparing (pleurectomy/decortication, P/D) (11–13). Surgery can be coupled with intraoperative treatments (14–17), but a general consensus on the proper multimodality approach is lacking.

### Radiotherapy

RT is used as an adjuvant or neoadjuvant treatment in MPM, mainly in a palliative setting (8–10). As standard practice, patients undergoing an EPP receive adjuvant conventionally fractionated RT (50–60 Gy) in the ipsilateral hemithorax area (18, 19). In node-negative MPM patients, neoadjuvant therapy, based on intensity-modulated RT (IMRT) consisting of a fractionated irradiation of 5–6 Gy, is delivered before EPP (20–22). In contrast, prophylactic radiotherapy of chest wall tracts after surgery to prevent parietal tumor seeding is not recommended anymore by the ASCO guidelines following the results of the SMART and PIT trials (23–25). Recently, adjuvant hemithoracic pleural RT has been shown to be effective and safe (26–28). Advanced RT treatments, e.g., proton therapy (29) or Arc therapy (a novel and accurate IMRT modality) (30), alone or combined with immunotherapies, are being tested to improve RT impact in MPM management.

### Angiogenesis Inhibitors

The angiogenic process plays an important role in MPM maintenance. VEGF receptor tyrosine kinase inhibitor (TKI) monotherapy yielded modest results (31–37). The addition of bevacizumab, a humanized monoclonal antibody against VEGF, to cisplatin-pemetrexed CT increases the median overall survival (OS) from 16.1 to 18.8 months and progression-free survival (PFS) from 7.3 to 9.2 months, as shown in the phase III MAPS study (NCT00651456) (38). Since this therapeutic regimen showed manageable toxicities, it has been included in the National Comprehensive Cancer Network guidelines (category 2A) (39), although it is not yet approved by the FDA or EMA. The SWOG S0905 phase I study evaluated the combination of cisplatin-pemetrexed CT with cediranib, a VEGF/PDGF receptor inhibitor, demonstrating a preliminary promising efficacy and reasonable toxicity profile (40) but, when compared to placebo, this combination failed to significantly increase OS and PFS in the following randomized phase II trial (41). Nintedanib is an inhibitor of three (triplet regimen) different growth factor receptors (VEGFR, PDFGR, and FGFR) and its administration in combination with CT improved the objective response rate (ORR) from 44 to 57% and the median PFS (9.7 vs. 5.7 months) compared to placebo in the LUME-Meso trial (42). Data from the phase III LUME-Meso trial (NCT01907100) have recently been published, and the primary PFS endpoint failed, not confirming the previous phase II trial results (43). Other TKIs, such as the anti-VEGFR axitinib (44) or the multi-target inhibitor of VEGFR1/2/3, FGFR-1, PDGFR-β, and RAF/cKit pathway sorafenib failed to improve median OS and PFS in chemonaive or CT-pretreated MPM patients (45, 46). The limited success of anti-angiogenic drugs is due to the lack of good predictive biomarkers to guide the selection of suitable patients for this therapy. Recently, blocking of FGF signaling has been pursued through the sequestration of FGFs with the GSK3052230 ligand trap molecule to avoid toxicities associated with FGFR inhibitors. A phase Ib study indicates that a combination of GSK3052230 plus cisplatin-pemetrexed-CT leads to an ORR of 44% and to a median PFS of 7.4 months with limited adverse events (47).

### Synthetic Lethal Therapies

Some MPM tumors cannot synthesize arginine due to the loss of argininosuccinate synthetase 1 (ASS1) gene expression. ASS1 deficiency is twice more frequent in the biphasic/sarcomatoid histotypes than in the epithelioid subtype. *In vitro* experiments suggest that depletion of arginine through exposure to a specific deaminase leads to synthetic lethality (48). The TRAP phase I trial (NCT02029690) demonstrated a positive effect of treatment with pegylated arginine deaminase (ADI-PEG 20) combined with CT in ASS1-deficient MPM patients (49). The ATOMIC-Meso phase III trial (NCT02709512) is recruiting patients with ASS1 gene loss. Genomic studies on MPM cells reported a reduced or absent expression of an enzyme involved in DNA repair and Ca^2+^-dependent apoptosis BAP1 in ~50% of sporadic MPMs. *In vitro* studies demonstrated that BAP1-mutated cells are less sensitive to ionizing radiation causing DNA double-strand breaks (50, 51) or to the DNA synthesis inhibitor gemcitabine (52), highlighting the contribution of BAP1 in DNA damage signaling and repair and a possible role as a predictive biomarker (53). Inherited loss-of-function mutations in BAP1 predispose to multiple carcinomas, including mesothelioma (54–56). Interestingly, MPM patients with germline mutated BAP1 or with genetic alterations in other DNA repair genes and treated with platinum CT showed a significantly longer median OS than patients devoid of the same mutations (57). Hence the BAP1 mutational status at diagnosis could be an important factor in predicting MPM patients' response to CT and may sensitize patients to synthetic lethality therapies that hit other components of the DNA repair machinery. Accordingly, as already suggested by Srinivasan et al. (58), the homologous repair (HR) component PARP-1 would be an excellent target for a synthetic lethality approach, given that MPM cells are frequently characterized by HR deficiency and unrepaired DNA damage accumulation due to the aforementioned BAP1 mutations. PARP-1 inhibitors, such as niraparib and olaparib, clearly decreased MPM cell survival, albeit regardless of BAP1 status. BAP1 loss also up-regulates the expression of EZH2, a Polycomb Repressive Complex-2 (PRC2) component involved in epigenetic silencing (59) and oncogenic pathways (60), suggesting sensitivity of BAP1-deficient MPM tumors to EZH2 inhibition. A phase II clinical trial (NCT02860286) is ongoing to evaluate the efficacy of the EZH2 inhibitor tazemetostat in MPM patients (61).

Finally, the synthetic lethality of inhibition of the Focal Adhesion Kinase (FAK) tyrosine kinase with loss of Merlin protein, the first involved in the survival, proliferation, and migration of tumor cells (62) and the second, a tumor suppressor encoded by the NF2 gene frequently mutated in MPM (5), has been proposed. Despite an encouraging positive trend observed in phase I trial in which FAK inhibitor GSK2256098 was tested in MERLIN-negative patients (63), a second large phase II trial (COMMAND, NCT01870609) demonstrated that neither PFS nor OS was improved by the FAK TKI defactinib as compared to placebo when administered as a maintenance treatment after frontline CT (64).

### Immunotherapies

Multiple lines of evidence point to the involvement of the immune system in the pathogenesis and sensitivity to therapy of MPM (65, 66). Spontaneous regressions in some patients are attributable to an activation of the immune system (67, 68). Moreover, B cells are essential for a good prognosis (69) in murine preclinical models of mesothelioma treated with immunotherapy, indicating that antibodies are generated and contribute to the therapeutic effect. Also, the presence of cytotoxic CD8^+^ tumor-infiltrating lymphocytes (TILs) is a good prognostic marker in MPM (70, 71).

MPM can be immunogenic but develops mechanisms to evade immune eradication. PD-L1 is the ligand for PD-1, a receptor expressed by activated T and B cells. Binding of PD-L1 to PD-1 affects effector T-cell and B-cell function and ultimately leads to exhaustion and apoptosis (72). Recently PD-L1 was shown to be expressed in 40% of MPMs, almost all of the sarcomatoid subtype, and was associated with a significantly poorer outcome, with a median survival of 5 months for PD-L1^+^ MPM patients vs. 14.5 months for PD-L1^−^ tumors (*p* < 0.0001) (73). However, PD-L1 expression is heterogeneous among MPM cells and could vary during treatment, limiting the efficacy of anti-PD-(L)1 therapy (74, 75).

### Immune Checkpoint Inhibitors

Immune checkpoint inhibitors (ICIs) are immune-modulating agents that boost the latent immune-response kept in check by the tumor. PD-1/PD-L1 and CTLA-4 inhibitory functions are targeted by immunomodulatory therapies, allowing T- and B-cell (re-)activation (76). Recently, many ICIs, including anti-CTLA-4, a glycoprotein expressed on regulatory and on activated CD4^+^ and CD8^+^ T cells, or anti-programmed death 1 (PD-1)/PD-L1 antibodies, have been approved for the treatment of solid and hematological malignancies (76–78).

Despite early enthusiasm for the results of tremelimumab, an anti-CTLA-4 ICI, as first-line therapy (79), its use as a second- or third-line treatment demonstrated no benefit of CTLA-4 inhibition over placebo (DETERMINE, NCT01843374) (80). Nivolumab efficacy was tested as a second- or third-line treatment alone vs. placebo in MPM patients in two recently completed phase II studies (NivoMes, NCT02497508, and MERIT, JapicCTI-163247) with ORRs of 24.0 and 29.4% and disease control rates (DCRs) of 50.0 and 67.6%, respectively (81, 82). A clear correlation between response and PD-L1 expression was reported (81). An ongoing randomized, placebo-controlled phase III trial is testing the efficacy of nivolumab in relapsed mesothelioma (CONFIRM, NCT03063450) (83).

The anti-PD-1 ICI pembrolizumab has been evaluated in different phase I (KEYNOTE-028, NCT02054806) and II (NCT02399371) studies as a second- or third-line treatment, showing promising DCR and prolonged disease stability (84–86). The results from the randomized phase III trial PROMISE-meso (NCT02991482) were instead disappointing, with relapsed MPM patients receiving pembrolizumab or single-agent CT failing to show an improved median OS and PFS despite a superior ORR for pembrolizumab compared to a CT regimen (22 vs. 6%) (87). Popat and colleagues suggest that ICI treatment should be tested at earlier stages and on patients that are better stratified to benefit from longer periods of immunotherapy.

Other ICIs, like the Inducible T-cell COStimulator (ICOS) agonist GSK3359609, alone or in combination with pembrolizumab, are being evaluated in advanced solid tumors including MPM (INDUCE-I, NCT02723955) (88).

### Combination Strategy

Two ICIs against different targets can be combined. An ipilimumab (anti-CTLA-4) and nivolumab (anti-PD-1) combination was tested in the phase-II MAPS2 trial (89) in relapsed MPM patients. The results indicated that the primary endpoint, DCR after 12 weeks, was reached by combined therapy (50%) and not by nivolumab alone (44%). An ORR of 25.9 vs. 18.5% and a modest increase of median response duration (7.4 vs. 7.9 months) were achieved in the combination and nivolumab groups, respectively. Severe treatment-related side effects were registered in 17% of patients. The same combination is being investigated in a randomized phase III trial (Checkmate 743, NCT02899299) in the front-line setting (90). Similarly, the combined therapy of tremelimumab plus durvalumab, an anti-PD-L1 antibody, tested in the phase II NIBIT-MESO-1 trial (NCT02588131), resulted in grade 3–4 treatment-related side effects in 17.5% of patients (91). A phase III study is evaluating the combination of pembrolizumab with pemetrexed and platinum-based CT vs. pembrolizumab or CT alone as first-line treatment for MPM patients (NCT02784171).

## Innovative Therapeutic Approaches for Malignant Mesothelioma

### miRNA Replacement

miRNA replacement is an innovative anti-cancer approach that restores miRNA expression by delivering miRNAs or miRNA mimics. Restored miRNAs can interfere with the expression of proteins endowed with oncogenic activity (92–94) thereby inhibiting proliferation or inducing apoptosis of tumor cells (95).

miR-16 is often downregulated in MPM, while its expression in *in vitro* and in murine xenografts results in decreased cell proliferation, decreased glucose uptake, and increased mortality (95). The feasibility of miR-16 exploitation by delivering its mimic encapsulated into an anti-EGFR-coated bacterially-derived shell termed EnGeneIC Dream Vector (TargomiR) (96) was shown in the NCT02369198 trial, which reported efficacy and good tolerability in patients with relapsed MPM (97). TargomiR therapy was associated with a drop in glucose uptake in 60% of patients as measured by PET-CT, while 73% of patients achieved disease control.

### Tumor Treating Fields

Recently, the FDA approved an innovative first-line treatment for MPM patients as a humanitarian use device, called NovoTTF-100L, that is based on the delivery of specific electric frequencies (Tumor Treating Fields, TTF) in combination with CT, to interfere with cancer cell proliferation. *In vitro* and *in vivo* data (98) are consistent with recent STELLAR phase II registration trial (NCT02397928) results, where a median OS of 18.2 months and low systemic toxicity have been experienced by the patients treated with TTF plus CT (99).

### Oncoviral Therapies

In the wake of successful phase I and II studies (100, 101), a phase III clinical trial (INFINITE, NCT03710876) is evaluating the efficacy of an Adenovirus-Delivered Interferon Alpha-2b (rAd-IFN) in combination with celecoxib and gemcitabine in MPM patients who failed previous regimens. A phase II study (NCT04013334) is testing the efficacy of Ad-SGE-REIC/MTG201, an adenoviral vector for the expression of Reduced Expression in Immortalized Cell (REIC)/Dickkopf-3 (Dkk-3) gene in combination with nivolumab. The Dkk-3 protein is a Wnt signaling pathway antagonist that induces cancer cell death and antitumor immune response. A previous phase I/II study showed that intrapleural virus administration was safe and well-tolerated and that Dkk-3 gene expression allowed durable disease control (102). Preclinical studies evaluated the replication-competent neuroattenuated Herpex Simplex Virus (HSV-1716) as oncolytic virotherapy for mesothelioma, showing cytotoxicity in combination with CT or RT *in vitro* and reduced tumor growth also at low doses *in vivo* in MPM murine models (103). The results of a phase I/IIa trial (NCT01721018) testing the intrapleural administration of HSV-1716 demonstrated virus replication, pleural Th1 cytokine response, and anti-tumor immunoglobulin production (104). The use of other viral vectors [reviewed in (105)], such as attenuated versions of vaccinia or measles virus genetically engineered to produce human thyroidal sodium iodine symporter (NIS), is being investigated in different phase I clinical trials (NCT02714374, NCT01503177).

### Dendritic Cell Vaccination

Cancer vaccines aim at inducing tumor-specific effector T cells that reduce tumor mass and induce tumor-specific memory T cells to curtail tumor relapse (106). Autologous dendritic cell vaccination (DCV) has shown efficacy in MPM treatment. The PMR-MM-002 clinical trial (NCT01241682) demonstrated the safety and feasibility of tumor lysate-pulsed dendritic cells as therapeutic adjuvants in MPM patients (107). The DENIM phase II/III randomized clinical trial (NCT03610360) will treat MPM patients with dendritic cell immunotherapy plus best supportive care (BSC) and compare the results with BSC alone (108). Other vaccination-based therapies currently under investigation are autologous DC loaded with Wilms' Tumor Antigen (WT1) (109) combined with CT (MESODEC, NCT02649829) and autologous TILs plus IL-2 (110). Based on the results obtained by PMR-MM-002 and by ICIs, a phase Ib MESOVAX clinical trial (NCT03546426) is recruiting MPM patients to test the efficacy of a tandem combination of autologous DCV and pembrolizumab at our institute.

### Mesothelioma Targeting Antigens

MSLN is a glycoprotein expressed more on the cell surface of several tumors, including MPM cells, than in normal tissues (111). A phase II clinical trial (NCT00738582) testing amatuximab, a chimeric anti-MSLN mAb, plus standard CT compared to CT alone showed a promising OS of 14.8 months (112) that was not confirmed in the ARTEMIS trial (NCT02357147). Anetumab ravtansine (AR), an anti-MSLN antibody conjugated with the cytotoxic anti-tubulin drug ravtansine, showed a 50% ORR and 90% DCR in pretreated patients (113). A second phase II randomized clinical trial showed that AR did not improve survival compared to the anti-mitotic chemotherapeutic, vinorelbine, as a single agent (114). The combined regimen of pembrolizumab plus AR will be evaluated in a phase 1/2 trial (NCT03126630) that is recruiting only MSLN-positive patients. A phase I study (NCT02798536) is currently active to assess a novel low-immunogenic anti-MSLN recombinant immunotoxin, RG778/LMB-100 (115), composed of a human single-chain variable fragment (scFv)-targeting moiety directed against MSLN linked to Pseudomonas exotoxin A (PE). The phase I trial NCT01675765 evaluated the sequential administration of the cancer vaccine CRS-207, an attenuated form of *Listeria monocytogenes* expressing MSLN, with or without cyclophosphamide followed by consolidation CT, to stimulate an innate and adaptive immunity against MSLN-expressing cells. The cyclophosphamide arm showed acceptable toxicity and a DCR of 89%, a PR of 54%, an SD of 29%, and a median PFS and OS of 7.5 and 14.7 months, respectively (116).

The success of advanced cell-based therapies, e.g., Chimeric Antigen Receptor-transduced T cells (CAR-T) in hematological tumors, awoke interest as well for MPM (117). CAR-T-cell receptors directed against MSLN are being investigated in several phase I clinical trials. The critical issues in Adoptive Cell Therapy (ACT) and CAR-T treatment are the safety profile and the degree of off-tumor toxicity. Intravenous or intra-tumor administration of MSLN-CAR-T cells (NCT01355965) (118) obtained by T-cell electroporation with encoding mRNA to achieve transient expression resulted in moderate responses and low toxicity (119). A phase I study (NCT02414269) drawing on preclinical results in orthotopic mouse MPM models (120, 121) is ongoing to test the MSLN-CAR-T cells in multi-treated MPM patients. Preliminary results presented at the AACR [Abstract CT036, (122)] and ASCO [Abstract 2511, (123)] meetings this year have shown an ORR and DCR of 36.8 and 57.8%, respectively, in a cohort treated off protocol in combination with pembrolizumab.

Fibroblast activation protein (FAP) is another interesting target expressed by all MPM subtypes and by cancer-associated fibroblasts (CAFs) and exploited by FAP-targeted CAR-T cells in an ongoing phase I trial (NCT01722149) (124) Preliminary results presented at the ESMO congress this year showed a good tolerance of treatment and persistence of CAR-T cells (125).

CD26 is a receptor overexpressed by all MPM histotypes and involved in immune regulation, T-cell activation, and the malignant potential of several cancers (126, 127). YS110 is a humanized mAb targeting CD26 that is currently under investigation in a phase I clinical trial (NCT03177668) in MPM patients. Preliminary results show that 50% (13/26) of patients achieved SD, with a median PFS of 43 days (128, 129).

## Discussion

**Figure 1:**
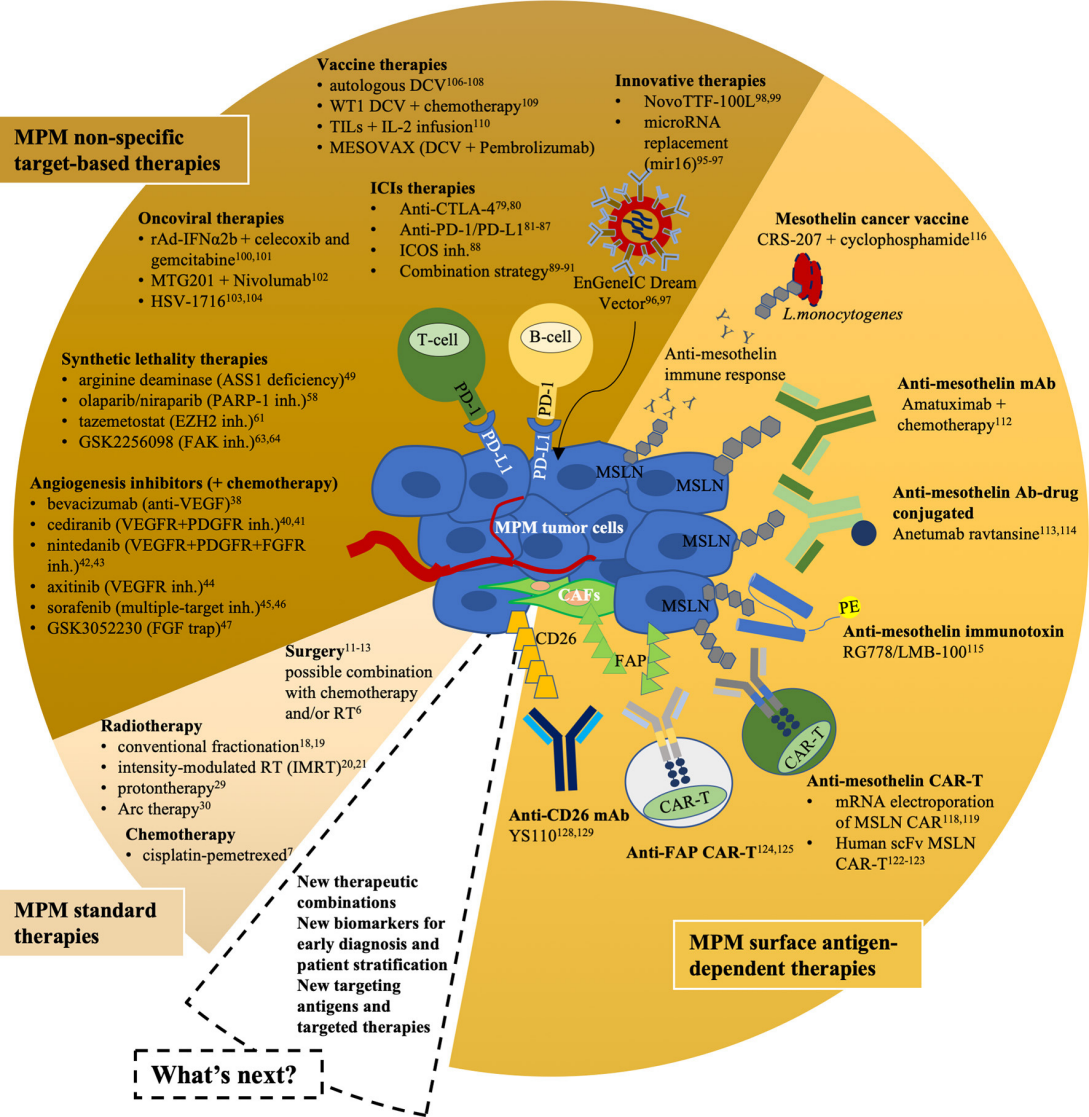
Current and innovative clinical approaches for MPM. Different segments represent MPM standard therapies (light brown), MPM non-specific target-based therapies (brown), and MPM surface antigen-dependent therapies (orange). inh., inhibitor; DCV, dendritic cell vaccination; WT1, Wilms' Tumor Antigen; CAR, chimeric antigen receptor; IL-2, interleukin-2; TILs, tumor-infiltrating lymphocytes; MSLN, mesothelin; FAP, fibroblast activation protein; CAFs, cancer-associated fibroblasts; PE, *Pseudomonas exotoxin* A.

Despite amazing efforts devoted to understanding and treating MPM better (Figure 1 and Table 1), clinical practice has not changed over the past decades, and CT remains the only standard option. Anti-angiogenic therapies and also ICIs that showed impressive clinical responses in other solid malignancies have little impact on survival in MPM as single agents, while ICI combination efficiency comes at the cost of relevant toxicities. The hopes for patients with MPM are, therefore, innovative therapies such as oncoviral, TTFields, TargomiRs, and CAR therapies in combination with anti-PD-1 ICIs that have shown good preliminary efficacy, although the results need confirmation in larger trials.

**Table 1:** Overview of MPM clinical trials.

**References**	**Clinical trial code**	**Acronymous**	**Type of study**	**Treatment**	**OS (months)**	**PFS (months)**	**ORR (%)**	**DCR (%)**	**Result or status**
**ANTI-ANGIOGENIC THERAPIES**									
Zalcman et al. (38)	NCT00651456	MAPS	III	CT -/+ bevacizumab	**18.8**	9.2	NE	NE	Pos
Tsao et al. (41)	NCT01064648	SWOG S0905	II	CT + ceradinib or pl.	10.0	**7.2**	50.0	NE	Neg
Scagliotti et al. (43)	NCT01907100	LUME-Meso	III	CT + nintedinab or pl.	14.4	**6.8**	45.0	91.0	Neg
Buikhuisen et al. (44)	NCT01211275	–	II	CT -/+ axitinib	18.9	5.8	36.0	79.0	Neg
Dubey et al. (45)	NCT00107432	–	II	Sorafenib	9.7	3.6	**6.0**	60.0	Neg
Papa et al. (46)	NCT00794859	SMS	II	Sorafenib	9.0	**5.1**	6.0	62.0	Neg
van Brummelen et al. (47)	NCT01868022	–	Ib	GSK3052230 + CT	NE	7.4	**39.0**	86.0	Pos
**SYNTHETIC LETHALITY THERAPIES**									
Beddowes et al. (49)	NCT02029690	TRAP	I	ADI-PEG 20 + CT	6.3	5.2	0	80.0	Pos (primary endpoints: recommended dose, safety, and tolerability)
	NCT02709512	ATOMIC-Meso	III	ADI-PEG 20	–	–	–	–	Ongoing
Zauderer et al. (61)	NCT02860286	–	II	Tazemetostat	NE	NE	NE	**51.0**	Pos
Fennell et al. (64)	NCT01870609	COMMAND	II	Defactinib or pl.	**12.7**	**4.1**	18.0	64.0	Neg
**IMMUNOTHERAPIES**									
Calabrò et al. (79)	NCT01649024	MESOT-TREM-2008	II	Tremelimumab	10.7	6.2	**7.0**	31.0	Neg
Maio et al. (80)	NCT01843374	DETERMINE	IIb	Tremelimumab or pl.	**7.7**	2.8	5.0	28.0	Neg
Quispel-Janssen et al. (81)	NCT02497508	NivoMes	II	Nivolumab	11.8	2.6	24.0	**47.0**	Pos
Okada et al. (82)	JapicCTI-163247	MERIT	II	Nivolumab	17.3	6.1	**29.4**	NE	Pos
Fennell et al. (83)	NCT03063450	CONFIRM	III	Nivolumab or pl.	–	–	–	–	Ongoing
Alley et al. (84)	NCT02054806	KEYNOTE-028	I	Pembrolizumab	18.0	5.4	**20.0**	72.0	Pos
Desai et al. (85)	NCT02399371	–	II	Pembrolizumab	11.5	4.5	**19.0**	66.0	Pos
Popat et al. (87)	NCT02991482	PROMISE-meso	III	Pembrolizumab vs. CT	10.7	2.5	22.0		Neg
Angevin et al. (88)	NCT02723955	INDUCE-I	I	GSK3359609	–	–	–	–	Ongoing
Scherpereel et al. (89)	NCT02716272	MAPS2	II	Nivolumab vs. nivolumab + ipilumab	11.9–15.9	4.0–5.6	19.0–28.0	**44.0**–**50.0**	Pos
Zalcman et al. (90)	NCT02899299	Checkmate 743	III	Nivolumab + ipilumab vs. CT	–	–	–	–	Ongoing
Calabrò et al. (91)	NCT02588131	NIBIT-MESO-1	II	Tremelimumab + durvalumab	16.6	5.7	**28.0**	63.0	Pos
–	NCT02784171	CCTG	III	CT vs. CT + pembrolizumab vs. pembrolizumab	–	–	–	–	Ongoing
**INNOVATIVE THERAPIES**									
van Zandwijk et al. (97)	NCT02369198	MesomiR 1	I	TargomiRs	6.7	NE	5.0	73.0	Pos (primary endpoints: MTD and DLT)
Ceresoli et al. (99)	NCT02397928	STELLAR	II	TTFields + CT	**18.2**	7.6	40.0	97.0	Pos
**ONCOVIRAL THERAPIES**									
Sterman et al. (101)	NCT01119664		I/II	–/+ CT + rAd-IFNa2b + CT	21.5	–	25.0	88.0	Pos (primary endpoint: safety)
	NCT03710876	INFINITE	III	rAd-IFNa2b + celecoxib + gemcitabine	–	–	–	–	Ongoing
Goto et al. (102)	UMIN000013568	–	I/II	Ad-SGE-REIC		3.4		62.0	Pos (primary endpoints: safety and tolerability)
–	NCT04013334	MTG201-MPM-001	II	Ad-SGE-REIC + nivolumab	–	–	–	–	Ongoing
Danson et al. (104)	NCT01721018	–	I/IIa	HSV-1716	15.0	NE	NE	50.0	Pos (primary endpoints: safety and tolerability)
–	NCT01503177	–	I	Measles virus encoding NIS	15.0	2.1	0	67.0	Pos (primary endpoint: AE profile)
**DENDRITIC CELL VACCINATION**									
Cornelissen et al. (107)	NCT01241682	PMR-MM-002	I	Tumor lysate-pulsed DCV	NE	NE	NE	80.0	Pos (primary endpoint: number of cytotoxic T cells and regulatory T cells in the blood of patients)
Belderbos et al. (108)	NCT03610360	DENIM	II/III	Tumor lysate-pulsed DCV + BSC vs. BSC	–	–	–	–	Ongoing
Berneman et al. (109)	NCT01291420	–	I/II	WT1 DCV	32.0	5.0	NE	NE	Pos (primary endpoint: immunogenicity of intradermal DCV)
–	NCT02649829	MESODEC	I/II	WT1 DCV + CT	–	–	–	–	Ongoing
Doherty et al. (110)	NCT02414945	TILs-003-Meso	I/II	TILs + IL-2	–	–	–	–	Ongoing
–	NCT03546426	MESOVAX	Ib	DCV + pembrolizumab	–	–	–	–	Ongoing
**ANTI-MSLN (IMMUNO)THERAPY**									
Hassan et al. (112)	NCT00738582	–	II	Amatuximab + CT	14.8	**6.1**	40.0	91.0	Neg
–	NCT02357147	ARTEMIS	II	Amatuximab + CT	–	–	–	–	Terminated for business reasons
Blumenschein et al. (113)	NCT01439152	–	I	AR	NE	NE	31.0	75.0	Pos (primary endpoint: MTD and pharmacokinetic profile)
Kindler et al. (114)	NCT02610140	–	II	AR or vinorelbine	10.1	**4.3**	8.0	NE	Neg
–	NCT03126630	MC1721	I/II	AR + pembrolizumab	–	–	–	–	Ongoing
–	NCT02798536	–	I	RG778/LMB-100 –/+ nab-paclitaxel	–	–	–	–	Ongoing
Hassan et al. (116)	NCT01675765	ADU-CL-02	I	CRS-207 –/+ cyclophosphamide + CT	14.7	7.5	54.0	89.0	Pos (primary endpoints: AE profile and induction of an immune response to MSLN)
Zhao et al. (118) and Beatty et al. (119)	NCT01355965	UPCC 17510	I	MSLN-CAR-T (mouse scFv)	NE	NE	NE	NE	Pos (primary endpoint: AE profile)
Adusumilli et al. (122, 123)	NCT02414269	–	I	MSLN-CAR-T (human scFv) + pembrolizumab	–	–	–	–	Ongoing
**IMMUNOTHERAPIES AGAINST NON-MSLN TARGETS**									
Curioni et al. (125)	NCT01722149	FAPME-1	I	FAP-targeted CAR-T	NE	NE	NE	NE	Pos (primary endpoint: safety)
Angevin et al. (128)	NCT03177668	YS1101	I	YS110 (anti-CD26)	**9.5**	**3.0**	**14.0**	**71.0**	Pos

*OS, overall survival; PFS, progression-free survival; ORR, objective response rate; DCR, disease control rate; CT, chemotherapy; pl., placebo; NIS, sodium/iodide symporter; DCV, dendritic cell vaccination; BSC, best supportive care; WT1, Wilms' Tumor Antigen; TILs, tumor-infiltrating lymphocytes; IL-2, interleukin-2; AR, Anetumab ravtansine; MSLN, mesothelin; CAR, chimeric antigen receptor; neg, negative; pos, positive; NE, not evaluated; MTD, maximum tolerated dose; DLT, dose-limiting toxicities; AE, adverse event; scFv, single chain fragment variable. Primary endpoints are in bold or indicated in the last column*.

## Author Contributions

FN prepared the manuscript and figure. MM prepared part of the manuscript, provided guidance to FN in preparing the manuscript, and proofread and edited the manuscript. MB, GB, AD, MG, and LC helped with the review and made vital modifications along with suggestions to improve the content. All authors contributed to manuscript revision, read, and approved the submitted version.

### Conflict of Interest

The authors declare that the research was conducted in the absence of any commercial or financial relationships that could be construed as a potential conflict of interest.
